# Kynurenic Acid Analog Attenuates the Production of Tumor Necrosis Factor-α, Calgranulins (S100A 8/9 and S100A 12), and the Secretion of HNP1–3 and Stimulates the Production of Tumor Necrosis Factor-Stimulated Gene-6 in Whole Blood Cultures of Patients With Rheumatoid Arthritis

**DOI:** 10.3389/fimmu.2021.632513

**Published:** 2021-04-09

**Authors:** Attila Balog, Borisz Varga, Ferenc Fülöp, Ildikó Lantos, Gergely Toldi, László Vécsei, Yvette Mándi

**Affiliations:** ^1^Department of Rheumatology and Immunology, University of Szeged, Szeged, Hungary; ^2^Institute of Pharmaceutical Chemistry and Research Group for Stereochemistry, Hungarian Academy of Sciences, University of Szeged, Szeged, Hungary; ^3^Department of Medical Microbiology and Immunobiology, University of Szeged, Szeged, Hungary; ^4^Department of Laboratory Medicine, Semmelweis University, Budapest, Hungary; ^5^Department of Neurology, University of Szeged, Szeged, Hungary

**Keywords:** kynurenine, TNF-α, TSG-6, TNFα-stimulated gene-6, calgranulins, HNP1–3, rheumatoid arthritis

## Abstract

**Objectives:** Rheumatoid arthritis (RA) is a chronic, inflammatory joint disease with complex pathogenesis involving a variety of immunological events. Recently, it has been suggested that kynurenic acid (KYNA) might be a potential regulator of inflammatory processes in arthritis. KYNA has a definitive anti-inflammatory and immunosuppressive function. The aim of the present study is to investigate the complex effects of a newly synthesized KYNA analog—SZR72 on the *in vitro* production of tumor necrosis factor-α (TNF-α), tumor necrosis factor-stimulated gene-6 (TSG-6), calprotectin (SA1008/9), SA100 12 (EN-RAGE), and HNP1–3 (defensin-α) in the peripheral blood of patients with RA and the various effects of the disease.

**Methods:** Patients with RA (*n* = 93) were selected based on the DAS28 score, medication, and their rheumatoid factor (RF) status, respectively. Peripheral blood samples from 93 patients with RA and 50 controls were obtained, and activated by heat-inactivated *S. aureus*. Parallel samples were pretreated before the activation with the KYNA analog N-(2-N, N-dimethylaminoethyl)-4-oxo-1H-quinoline-2-carboxamide hydrochloride. Following the incubation period (18 h), the supernatants were tested for TNF-α, TSG-6, calprotectin, S100A12, and HNP1–3 content by ELISA.

**Results:** SZR72 inhibited the production of the following inflammatory mediators: TNF-α, calprotectin, S100A12, and HNP1–3 in whole blood cultures. This effect was observed in each group of patients in various phases of the disease. The basic (control) levels of these mediators were higher in the blood of patients than in healthy donors. In contrast, lower TSG-6 levels were detected in patients with RA compared to healthy controls. In addition, the KYNA analog exerted a stimulatory effect on the TSG-6 production *ex vivo* in human whole blood cultures of patients with RA in various phases of the disease.

**Conclusion:** These data further support the immunomodulatory role of KYNA in RA resulting in anti-inflammatory effects and draw the attention to the importance of the synthesis of the KYNA analog, which might have a future therapeutic potential.

## Introduction

Rheumatoid arthritis (RA) is a chronic, inflammatory joint disease of autoimmune nature. The pathogenesis of the disease is complex, involving both immunological and genetic factors (1). RA is a systemic disease, but a variety of immunological events may occur outside the joint (2). Cytokines are known to have an established role in the disease pathogenesis. Pro-inflammatory cytokines, such as tumor necrosis factor-α (TNF-α), IL-1, and IL-17, provoke and maintain inflammation as well as bone and cartilage degradation (3). It is plausible and noteworthy that anti-cytokine agents seem to emerge as potent biological active molecules in the treatment of RA (4, 5).

Recently, it has been suggested that kynurenic acid (KYNA) might be a potential regulator of inflammatory processes in arthritic joints (6). Moreover, in a recent study, it has been observed that kynurenine prevents the development of the disease, while inhibition or deletion of indoleamine 2, 3-dioxygenase 1 (IDO1) has increased its severity (7).

**Figure 1:**
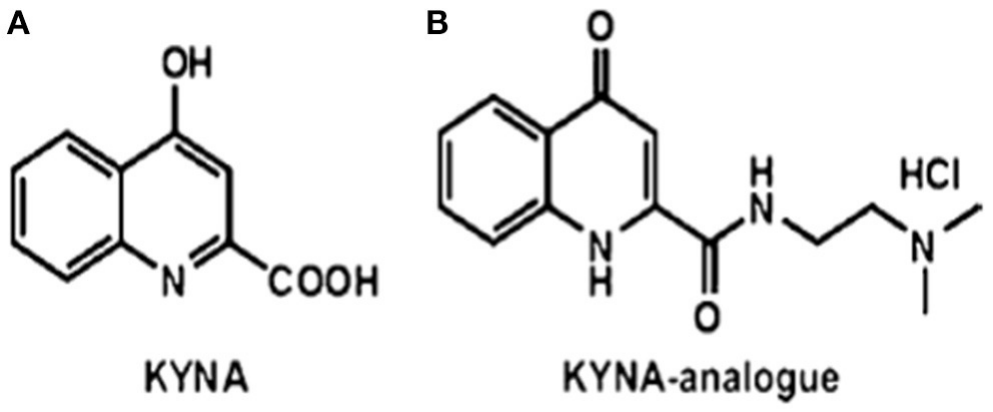
The structures of **(A)** kynurenic acid (KYNA) and **(B)** of the KYNA analog 2-(2-N,N-dimethylaminoethylamine-1-carbonyl)-1H-quinolin-4-one hydrochloride (18, 19) SZR72.

Kynurenic acid is a product of the kynurenine pathway of tryptophan metabolism (8). KYNA is an antagonist of ionotropic glutamate receptors, such as N-methyl-D-aspartate (NMDA) (9) and the α7 nicotinic acetylcholine receptor (α7nAchR) (10, 11), and it exerts neuroprotective impacts (12, 13). In addition, KYNA has a definitive immunomodulatory function (14, 15) exerting anti-inflammatory and immunosuppressive effects. These immunomodulatory properties are based on the signaling by G-protein-coupled receptor 35 (GP35) and aryl hydrocarbon receptor (ARH)-mediated pathways (16, 17). Previously, it has been reported that the newly synthesized analog of KYNA, such as 2-(2-N,N-dimethylaminoethylamine-1-carbonyl)-1H-quinolin-4-one hydrochloride—SZR72 (Figure 1) (18, 19) reduces TNF-α expression and secretion in human monocytes (20). Likewise, the KYNA analog has been shown to inhibit the secretion of α-defensin, such as HNP1–3 in human granulocyte cultures (20).

The chemical attributes of KYNA and SZR-72 are different, possibly influencing their ability to cross through the BBB (blood brain barrier) and membranes. There is evidence that KYNA could only poorly cross the BBB, while SZR-72 is BBB-permeable due to a water-soluble side chain with an extra cationic center (9, 18, 21). Furthermore, a facilitated membrane crossing of SZR-72 is suspected, which could influence intracellular signaling, including the activation of antioxidative and anti-apoptotic pathways (22). On the other hand, a distinct binding to the glycine site of the NMDA receptor has a higher affinity to SZR72 than KYNA (22).

Considering all of the above, the aim of our present study is to investigate the complex effects of a newly synthesized KYNA analog—SZR72 on the *in vitro* production of TNF-α, calprotectin (SA1008/9), SA100-12 [extracellular newly identified receptor for advanced glycation end-products binding protein (EN-RAGE)], and HNP1–3 (defensin-α) in the peripheral blood of patients with RA. Previously, it has been proven that the suppressive effect of the KYNA analog was more potent than that of an equimolar concentration of KYNA itself (20); therefore, this was used in the present study. These experiments were supplemented by measuring the effect of the KYNA analog on the tumor necrosis factor-stimulated gene-6 protein TSG-6 (TSG-6) concentrations in the human blood samples, since an opposite effect of KYNA on the TSG-6 production has formerly been observed (23).

The role of TNF-α is widely characterized in the pathogenesis of rheumatoid arthritis (RA) (4). Leukocyte activation and infiltration are critical events in the pathogenesis of RA. Relatedly, the role of calgranulins in the pathogenesis, diagnosis, and monitoring of rheumatic diseases has gained great attention in recent years (24, 25). Calgranulins are represented by the S100 protein family including S100A8, S100A9, and S100A12 (26). The S100A8 and S100A9 complexes—as calprotectins—are found in granulocytes and monocytes. S100A12 (EN-RAGE) is restricted mainly to granulocytes (27, 28).

Human neutrophil peptide 1–3 (HNP1–3), also known as defensin-α, may be secreted and released into the extracellular milieu during an inflammatory response following the activation of polymorphonuclear neutrophils during inflammation (29, 30). The defensin-α not only plays a role in microbial killing, but also in immunomodulation during inflammatory processes (31). The elevation of HNP1–3 has also been reported in patients with RA (32, 33). Similarly, the so-called calgranulins—calprotectin and S100A12 (EN-RAGE)—correlated with the clinical status of patients with RA (24, 34). These data draw the attention to the role of these inflammatory mediators, as alarmins in the development of RA.

## Materials and Methods

### Patients

Rheumatoid arthritis was classified according to the 2010 American College of Rheumatology (ACR)/European League Against Rheumatism (EULAR) classification criteria for RA (35). The detailed patient characteristics and clinical data are presented in Table 1. Patients with RA (*n* = 93) were grouped based on disease activity score in 28 joints (DAS28) of ≤2.6, 2.6 ≤ 3.2, 3.2 ≤ 5.1, and ≥5.1 remission (*n* = 30), mild (*n* = 18), moderate (*n* = 27), and severe (*n* = 18), respectively. Patients with RA were treated with biological response modifiers as anti-TNF therapy (*n* = 29), IL-6R antagonist (*n* = 10), rituximab (*n* = 4), abatacept (*n* = 1), tofacitinib (*n* = 1), or with conventional disease-modifying antirheumatic drugs (DMARDs), including methotrexate (*n* = 58), leflunomide (*n* = 7), sulfosalazine (*n* = 3), chloroquine (*n* = 5), and low dose methyl-prednisolone (*n* = 27). Anti-citrullinated protein/peptide antibody (ACPA) was measured using the ELISA-based routine laboratory methods with specificity to mutated citrullinated vimentin (MCV). Patients with RA (*n* = 93) were selected based on medication and their rheumatoid factor status (RF), respectively.

**Table 1:** Clinical characteristics of healthy individuals and RA patients.

**Characteristics**	**Healthy individuals *n* = 50**	**RA remission *n* = 30**	**RA mild *n* = 18**	**RA moderate *n* = 27**	**RA active *n* = 18**
Age, years	58 [46–67]	60 [53–67]	67 [59–73]	63 [55–73]	64 [56–74]
Gender, male/female	8/42	5/25	2/16	7/20	6/12
RA duration, years	–	16 [4–19]	11 [3–19]	8 [1–12]	1.5^b^^,^^c^ [0.33–9]
Rheumatoid factor (U/mL)	–	258 [92–500]	84 [19–428]	94 [42–468]	181 [17–500]
Anti-MCV (U/mL)	–	309 [53–999]	90 [24–825]	158 [55–−895]	83 [17–491]
CRP (mg/L)	BLD [BLD−2.3]	BLD [BLD−3.9]	3.6 [BLD−6.5]	11.4^a^^,^^b^^,^^c^ [7.3–28]	23^a^^,^^b^^,^^c^ [8.5–66]
ESR (mm/h)	7.5 [5–9]	11.5 [7–22]	25^a^ [12–44]	38^a^^,^^b^ [26–58]	38^a^^,^^b^ [25–78]

*Data are expressed as median [interquartile range]. Comparisons were made using the Kruskal–Wallis test*.

a p < 0.05 vs. healthy individuals,

b p < 0.05 vs. RA remission,

c *p < 0.05 vs. RA mild. BLD, below the level of detection (<2 mg/L)*.

As a further control group (healthy controls), we enrolled 50 age- and gender-matched healthy volunteers. All of them had a negative history of RA symptoms and a negative status upon detailed physical and laboratory examinations, including normal CRP and ESR values. The project was approved by the Ethics Committee of the University of Szeged (ETT-TUKEB 905/PI/09 and 149/2019-SZOTE). This study was conducted in full accordance with the Declaration of Helsinki (1964). The patients/participants provided their written informed consent to participate in this study.

### KYNA Analog SZR 72

KYNA amide (Figure 1) was designed in the Department of Pharmaceutical Chemistry and MTA-SZTE Research Group for Stereochemistry, University of Szeged, Szeged, Hungary. The synthesis was performed by the coupling of KYNA and 2-dimethylaminoethylamine, afterwards, treatment of ethanolic hydrogen chloride, resulting in N-(2-N, N-dimethylaminoethyl)-4-oxo-1H-quinoline-2-carboxamide hydrochloride (18, 19, 21).

### Human Blood Incubation Method

Ethylenediaminetetraacetic acid (EDTA)-anticoagulated peripheral blood samples from 93 patients with RA and 50 healthy controls were obtained. The samples (1 ml each) were incubated for 18 h in Heracell CO_2_ incubator (Thermo Fischer Scientific, MA, USA) at 37°C under controlled conditions, or in the presence of heat-inactivated *S. aureus* for 18 h (10^7^/m) as a TNF inducer (36). Parallel blood samples were pretreated before activation for 30 min with the KYNA analog at a concentration of 500 μ. SZR72 was freshly dissolved in phosphate buffered saline (PBS), thereafter diluted in Roswell Park Memorial Institute (RPMI) medium (SIGMA), and added in 100 μl volume to the blood sample. All other samples were supplemented thereafter with 100 μl RPMI medium to equalize the volumes. This concentration of SZR72 was considered optimal for the experiments performed previously (20, 23). Following the incubation period, the blood samples were centrifuged at 3,000 g, and the supernatants were tested for TNF-α (SIGMA, St. Louis, USA), TSG-6 (Fine Biotech, Wuhan, China), calprotectin (Hycult-Biotech, HK373-02, Uden, the Netherlands), S100A12 (CircuLex CY-8058 V2) (MBL International Corporation, MA, USA), and HNP1–3 (Hycult-Biotech HK324, Uden, the Netherlands) content by ELISA according to the instructions of the manufacturers. For the experiments performed with the human blood, we gained the approval of the ethics committee of the Medical Faculty of the University of Szeged, Szeged, Hungary (ETT-TUKEB 905/PI/09 and 149/2019 SZOTE). This study was conducted in full accordance with the Declaration of Helsinki (1964). The patients/participants provided their written informed consent to participate in this study.

### Statistics

Comparing different groups of patients and healthy controls, the Kruskal–Wallis test was applied. For the comparison of the concentrations of mediators within one group of patients, the Friedman test was used with the Dunn's post-test.

The *p* < 0.05 were considered significant. All statistical calculations were performed with the Graph-Pad Prism 5 statistical program (Graph Pad Software Inc., San Diego, CA, USA).

## Results

### KYNA Analog, SZR72 Attenuates TNF-α Production in the Human Whole Blood of Healthy Controls and of Patients With RA Stimulated by Heat-Inactivated *Staphylococcus aureus*

**Figure 2:**
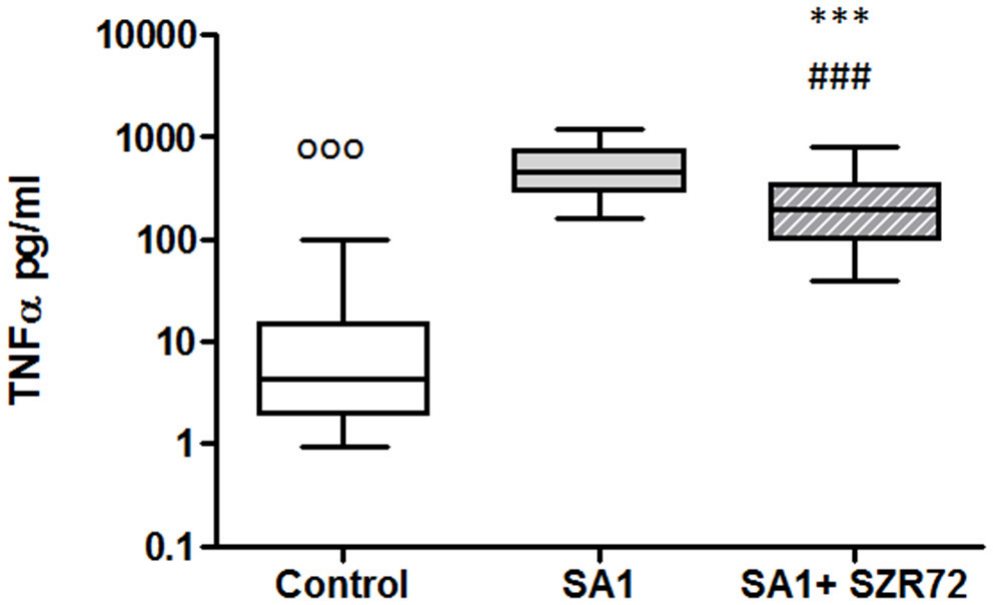
KYNA analog, SZR72 attenuates tumor necrosis factor-α (TNF-α) production in whole blood cells of normal controls stimulated by heat-inactivated *Staphylococcus aureus*. Ethylenediaminetetraacetic acid (EDTA)-anticoagulated blood samples of 1–1 ml from each of 50 donors were incubated with SZR72 at a concentration of 500 μm for 30 min prior to the addition of heat-inactivated *Staphylococcus aureus* (10^7^/ml). The concentrations of TNF-α in the plasma were determined after 18 h-incubation period. The data are depicted as box and whiskers plots, where the lines inside the boxes denote medians, and the boxes mark the interval between 25 and 75 percentiles, and the whiskers, the maximum and minimum. Significance was determined by the Friedman's test followed by Dunn's post-test. In Friedman'd test, *p* < 0.0001; ^ooo^*p* < 0.001 in control vs. SA1, ^###^*p* < 0.001 in control vs. SA1 + SZR72, ***p < 0.001 in control vs. SA1.

**Figure 3:**
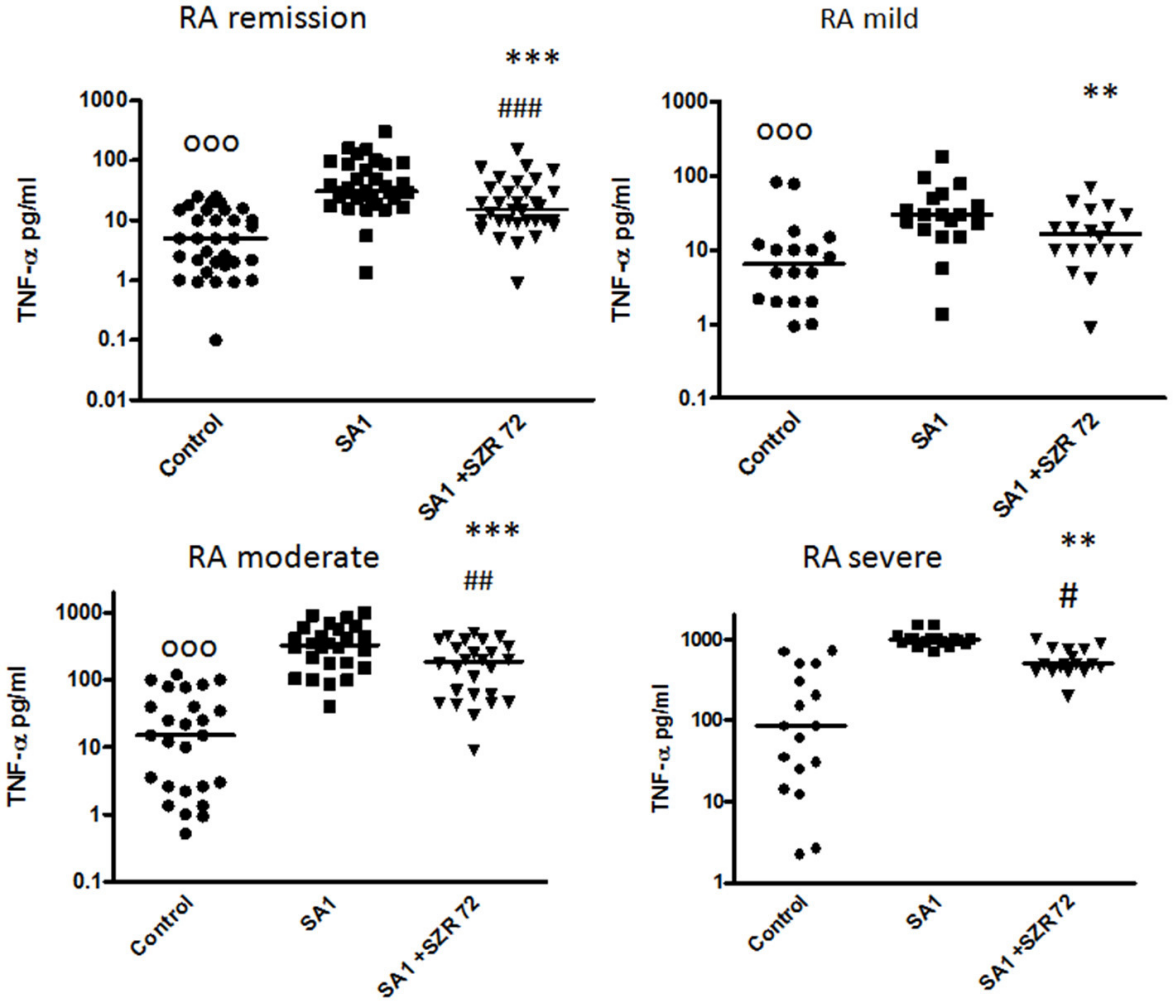
The effect of SZR72 on tumor necrosis factor-α (TNF-α) production in human whole blood cells of various groups of patients with RA stimulated by heat- inactivated *Staphylococcus aureus*. Each dot represents the individual value for one subject, and the horizontal lines denote the medians. Significance was determined by the Friedman's test and Dunn's post-test. Remission group: *n* = 30, *p* < 0.0001; control vs. SA1, significant, ^ooo^*p* < 0.001; control vs. SA1 + SZR72, ^###^*p* < 0.001; SA1 vs. SA1 + SZR72, ***p < 0.001 according to the Dunn's post-test. Mild group: *n* = 18, *p* < 0.0001; control vs. SA1, ^ooo^*p* < 0.001; control vs. SA1 + SZR72, non-significant; SA1 vs. SA1 + SZR72, **p < 0.01 according to the Dunn's post-test. Moderate group: *n* = 27, *p* < 0.0001; control vs. SA1, ^ooo^*p* < 0.001; control vs. SA1 + SZR72, ^##^*p* < 0.01; SA1 vs. SA1 + SZR72, ***p < 0.001. Severe group: *n* = 18, control vs. SA1, ^ooo^*p* < 0.001; control vs. SA1 + SZR72, ^#^*p* < 0.05; SA1 vs. SA1 + SZR72, **p < 0.01 according to the Dunn's post-test.

In pilot experiments, we investigated the effect of SZR72 on TNF-α production in healthy human blood donors (Figure 2). During the course of these experiments, 500 μm concentrations of SZR72 were applied; hence in previous experiments, this concentration proved to be the most effective (20, 23). There was a significant decrease in the in *vitro* TNF-α concentration in the supernatants of SA1-induced blood samples following SZR72 treatment. The TNF-α concentration in the basal (control) levels of blood cultures was between 0.9 and 99.5 pg/ml with the median of 4.35 pg/ml, and in the supernatants, the SA1-induced blood cultures were between 160 and 1,200 pg/ml, with a median of 450 pg/ml. SZR72 resulted in a significant decrease in TNF-α concentration in SA1-induced blood samples to 200 ng/ml as a median (Figure 2). Thereafter, we investigated to find whether the TNF-α production and the effect of SZR72 on RA was different in the various groups of the disease. Therefore, we stratified the patients according to their clinical status, mild (*n* = 18), moderate (*n* = 27), and severe (*n* = 18) status, or in the remission phase (*n* = 30) of the disease. The inhibitory effect of SZR72 on the SA1-mediated TNF-α production was different in various groups of patients (Figure 3). The highest concentrations were observed among patients with severe phase of the disease (basal/control median level at 85 pg/ml; SA1-induced median level at 995 pg/ml). The KYNA analog, SZR72 suppressed the TNF-α level of SA1-induced blood cultures to 500 pg/ml as a median; it was significant with the Friedman's test and with Dunn's post-test (Figure 3). Similarly, SZR72 significantly inhibited The SA1-induced TNF-α production in the remission group, and also in the mild and moderate groups of patients (Figure 3). The lowest basal/control TNF-α levels in the whole blood supernatants were measured in the plasma samples of patients with RA with the remission form of the disease [basal level with a median of 5 pg/ml; SA1-induced concentration at a median level of 30 pg/ml, and SZR72 suppressed it to 15 pg/m as a median (Figure 3)].

### The Effect of KYNA Analog SZR72 on TSG-6 Production in Human Whole Blood of Healthy Controls and of Patients With RA Stimulated by Heat-Inactivated *Staphylococcus aureus*

To ascertain whether the effects of the KYNA analog on the TNF-α production might be influenced by the increased induction of TSG-6, the concentrations of TSG-6 in whole blood cultures were determined in 93 patients with RA comparing them with normal control blood donors.

**Figure 4:**
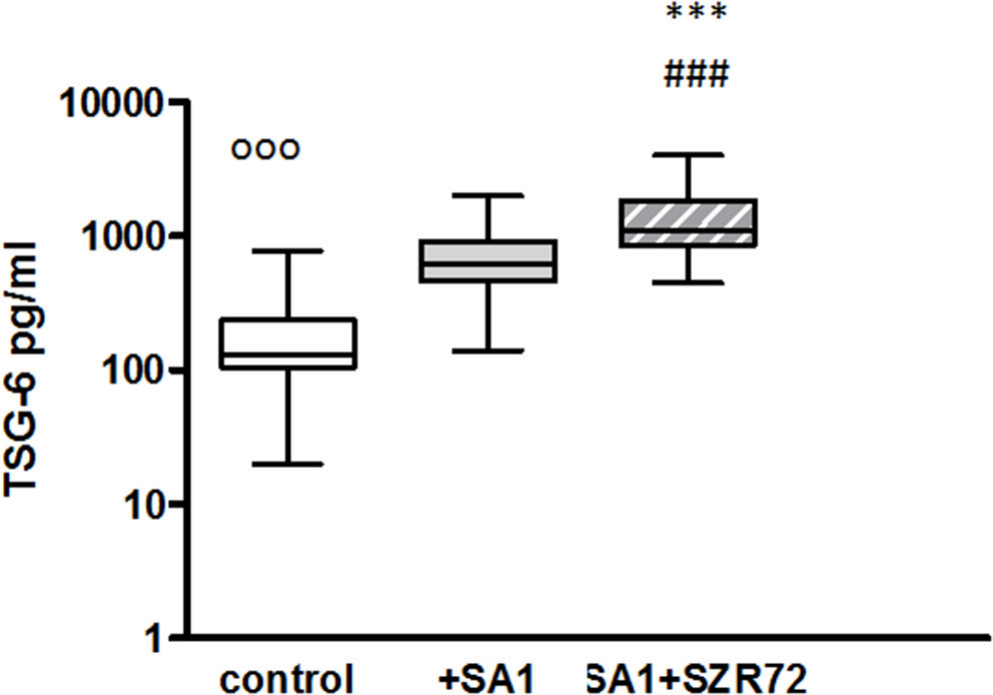
The effect of KYNA analog, SZR72 on TSG-6 production in human whole blood cells of normal donors stimulated by heat-inactivated *Staphylococcus aureus*. Ethylenediaminetetraacetic acid (EDTA)-anticoagulated blood samples 1–1 ml from each of 50 healthy donors were incubated with SZR72 at a concentration of 500 μm for 30 min prior to the addition of heat-inactivated *Staphylococcus aureus* (10^7^/ml). The concentrations of TSG-6 in the plasma were determined after an 18 h-incubation period. The data are depicted as box and whiskers plots, where the lines inside the boxes denote medians, and the boxes mark the interval between 25 and 75 percentiles, and the whiskers, the maximum and minimum. Significance was determined by the Friedman's test and Dunn's post-test. Control vs. SA1, ^ooo^*p* < 0.001; control vs. SA1 + SZR72, ^###^*p* < 0.001; SA1 vs. SA1 + SZR72, ***p < 0.001.

The TSG-6 level in the supernatants of basal/control blood samples of healthy subjects were elevated following the incubation with SA1, and it was further increased when 500 um SZR72 were added to the blood cultures (Figure 4). At a concentration of 500 μm, the KYNA analog SZR72 increased the TSG-6 level significantly in SA1-induced blood samples, with the median of 1,100 vs. 625 pg/ml, respectively, where *p* < 0.001 according to the Friedman's test (Figure 4). These experiments obtained with 500 μm of KYNA analog support our previous results (23) demonstrating the effects of KYNA and KYNA analogs on the TSG-6 RNA expression and the elevation of TSG-6 protein level.

**Figure 5:**
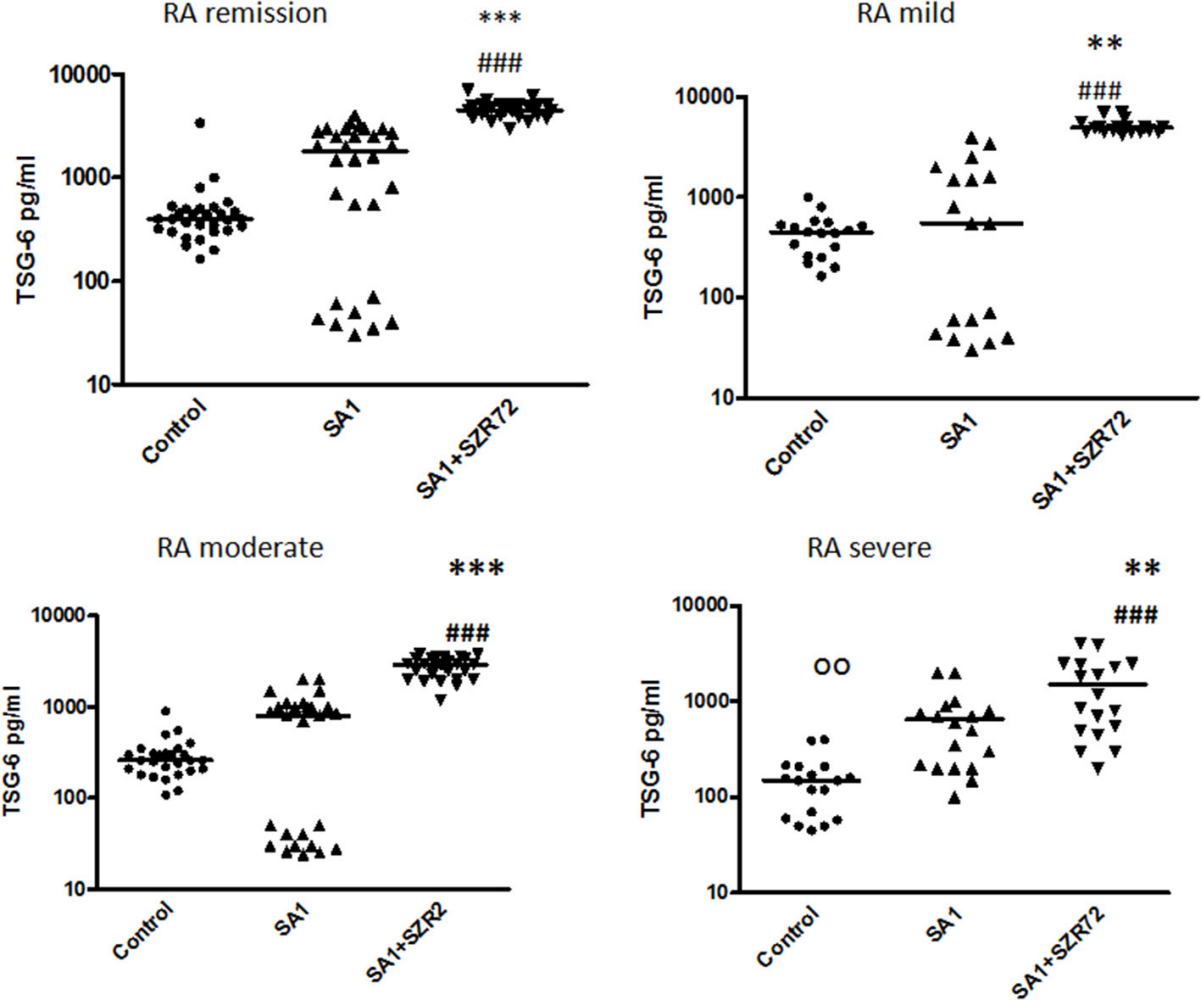
The effect of SZR72 on TSG-6 production in human whole blood cells of various groups of patients with RA stimulated by heat-inactivated *Staphylococcus aureus*. Each dot represents the individual value for one subject, and the horizontal lines denote the medians. Significance was determined by the Friedman's test and Dunn's post-test. Remission group: *n* = 30, *p* < 0.0001; control vs. SA1, non-significant; control vs. SA1 + SZR72, ^###^*p* < 0.001; SA1 vs. SA1 + SZR72, ***p < 0.001 according to the Dunn's post-test. Mild group: *n* = 18: *p* < 0.0001; control vs. SA1, non-significant; control vs. SA1 + SZR72, ^###^*p* < 0.001; SA1 vs. SA1 + SZR72, **p < 0.01. Moderate group: *n* = 27, control vs. SA1, non-significant; control vs. SA1 + SZR72, ^###^*p* < 0.001; SA1 vs. SA1 + SZR72, ***p < 0.001. Severe group: *n* = 18: control vs. SA1, ^oo^*p* < 0.01; control vs. SA1 + SZR72, ^###^*p* < 0.001; SA1 vs. SA1 + SZR72, **p < 0.01.

Next, we investigated to find whether the TSG-6 production and the effect of SZR72 on it was different in the various groups of the patients. Patients were categorized according to their clinical status: mild (*n* = 18), moderate (*n* = 27), and severe (*n* = 18), or being in the remission phase (*n* = 30) of the disease. The TSG-6 production of the SA1-induced samples was increased following SZR72 treatment in all groups of patients (Figure 5). Interestingly but not surprisingly, the lowest concentrations were observed in patients in the severe phase of the disease (basal/control median level at 150 pg/ml; SA1-induced median level at 550 pg/ml). The KYNA analog, SZR72 increased the TSG-6 level of SA1-induced blood cultures to 1,025 pg/ml as a median (Figure 5). On the contrary, the highest basal TSG-6 levels in the whole blood supernatants were measured in the plasma samples of patients with RA in the remission form of the disease; the basal level of TSG-6 showed a median of 400 pg/ml, which was induced to 1,800 pg/ml by SA1, and was further increased significantly by 500 μm SZR72 to 4,500 pg/ml (Figure 5). Similar results were obtained by analyzing the patients in the mild phase of the disease: basal level with a median of 445 pg/ml, SA1-induced concentration at a median level of 550 pg/ml, and SZR72 were increased to 4,950 pg/ml as a median.

### The Effect of KYNA Analog, SZR72 on Calprotectin Production in the Human Whole Blood of Healthy Controls and of Patients With RA Stimulated by Heat-Inactivated *Staphylococcus aureus*

The role of calprotectin in the pathogenesis, diagnosis, and monitoring of RA has gained great attention in recent years (24, 25). Therefore, we investigated the effects of SZR 72 and KYNA analog on the production of calprotectin *in vitro* in the whole blood.

**Figure 6:**
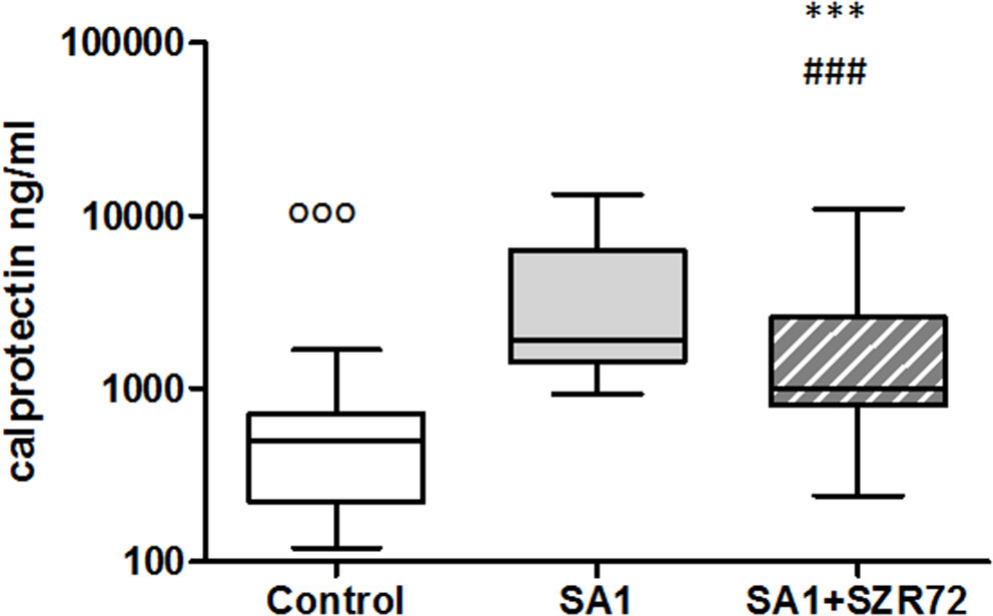
The effect of KYNA analog, SZR72 on calprotectin production in human whole blood cells of normal donors stimulated by heat-inactivated *Staphylococcus aureus*. Ethylenediaminetetraacetic acid (EDTA)-anticoagulated blood samples 1–1 ml from each of 50 normal blood donors were incubated with SZR72 at a concentration of 500 μm for 30 min prior to the addition of heat-inactivated *Staphylococcus aureus* (10^7^/ml). The concentrations of calprotectin in the plasma were determined after an 18 h-incubation period. The data are depicted as box and whiskers plots, where the lines inside the boxes denote medians, and the boxes mark the interval between 25 and 75 percentiles, and the whiskers, the maximum and minimum. Significance was determined by the Friedman's test. Control vs. SA1, ^ooo^*p* < 0.001; control vs. SA1 + SZR72, ^###^*p* < 0.001; SA1 vs. SA1 + SZR72, ***p < 0.001, according to the Dunn's post-test.

**Figure 7:**
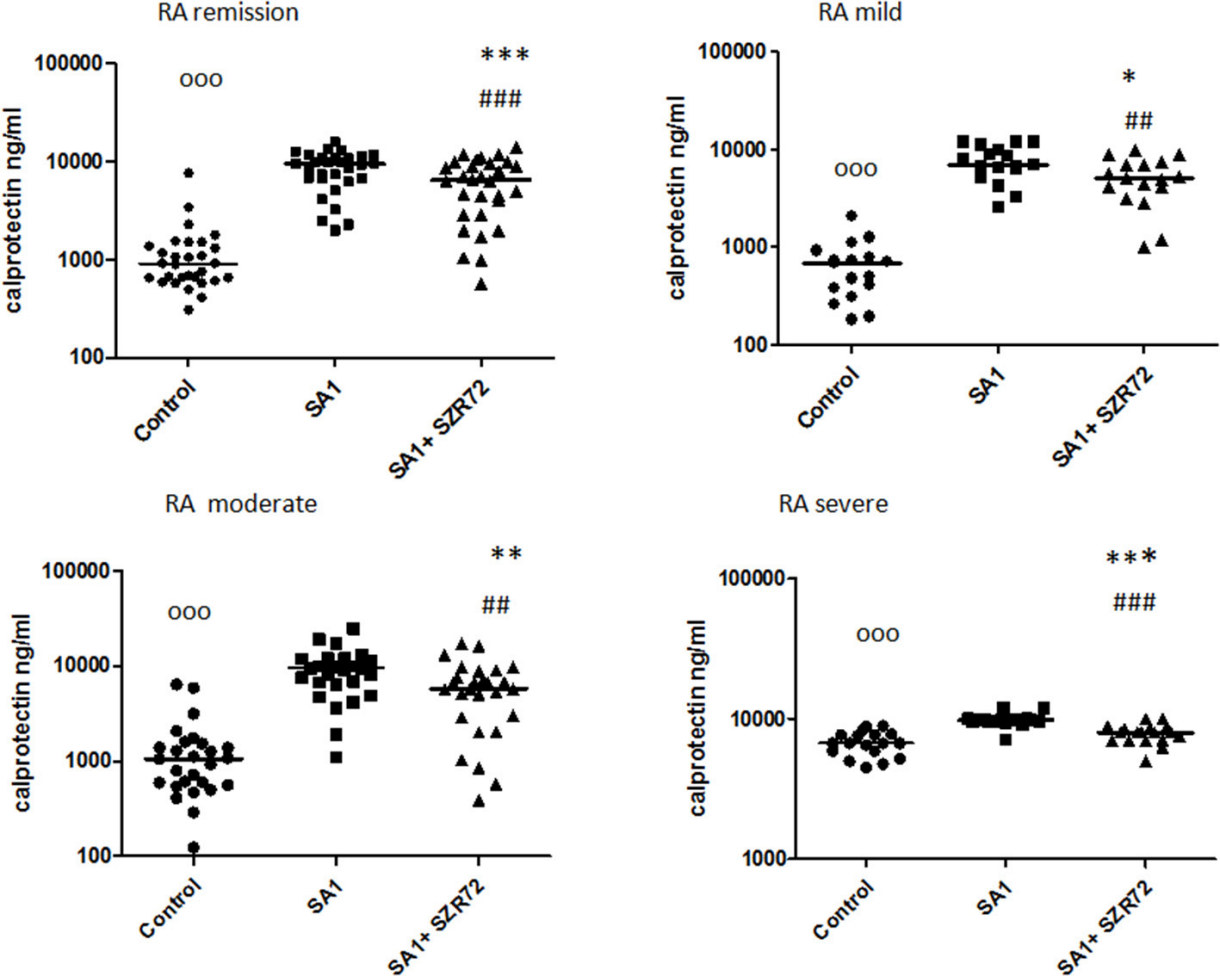
The effect of SZR72 on calprotectin production in human whole blood cells of various groups of patients with RA stimulated by heat-inactivated *Staphylococcus aureus*. Each dot represents the individual value for one subject, and the horizontal lines denote the medians. Significance was determined by the Friedman's test. Remission group: *n* = 30, *p* < 0.0001; control vs. SA1, ^ooo^*p* < 0.001; control vs. SA1 + SZR 72, ^###^*p* < 0.001; SA1 vs. SA1 + SZR72, ***p < 0.001 according to the Dunn's post-test. Mild group: *n* = 18, *p* < 0.0001; control vs. SA1, ^ooo^*p* < 0.001; control vs. SA1 + SZR72, ^##^*p* < 0.01; SA1 vs. SA1 + SZR 72, *p < 0.05 according to the Dunn's post-test. Moderate group: *n* = 27, *p* < 0.0001; control vs. SA1, ^ooo^*p* < 0.001; control vs. SA1 + SZR 72, ^##^*p* < 0.001; SA1 vs. SA1 + SZR 72, **p < 0.01 according to the Dunn post's test. Severe group: *n* = 18, *p* < 0.0001; control vs. SA1, ^ooo^*p* < 0.001; control vs. SA1 + SZR72, non-significant; SA1 vs. SA1 + SZR72, ***p < 0.001 according to the Dunn's post-test.

The concentrations in the basal levels of normal control blood cultures were between 120 and 1,680 ng/ml with the median of 500 ng/ml (Figure 6). The SA1-induced calprotectin production in whole blood cultures is as follows: the calprotectin concentrations in the supernatants in SA1-induced blood cultures varied from 930 to 13,300 ng/ml, with a median of 1,900 ng/ml. At a concentration of 500 μm, SZR72 suppressed the calprotectin level significantly in the *S. aureus*-activated blood cultures at a median of 1,000 ng/ml, where *p* < 0.01 with Friedman's test and Dunn's post-test (Figure 6). We investigated to find whether the calprotectin production and the effect of SZR72 were different in the various groups of the disease. Therefore, we grouped the patients according to their clinical status as mild (*n* = 18), moderate (*n* = 26), and severe (*n* = 18), or being in remission phase (*n* = 33) of the disease. The inhibitory effect of SZR72 on the SA1-mediated calprotectin production was observed in all groups of patients, but at a different level (Figure 7). As it was expected, the lowest levels were detected in the blood samples of patients with RA with a remission and mild state of the disease. Among the remission group, the median was 911 ng/ml, which was induced by SA1 (9,509 ng/mL) and SZR72 inhibited it to 6,520 ng/ml as a median *p* < 0.001 according to the Friedman's test. Similar results were observed concerning the basal levels of calprotectin among patients with the mild phase of the disease; their basal level was measured as 680 ng/ml as median, which was induced by SA1 to 7,000 ng/ml, and was decreased to 5,100 ng/ml as median following an incubation period of 18 h with SZR 72 (Figure 7). In contrast, the basal/control median level of calprotectin among patients with a severe status of disease was 6,700 ng/ml, which was increased to 9,800 ng/ml following the induction with SA1, and it was decreased to 800 ng/ml as a median as a result of treatment with 500 μm SZR72 (Figure 7). An intermediate basal level of calprotectin in patients with moderate form of disease was found to be at 1,050 ng/ml, and it was induced with *Staphylococcus aureus* to 9,500 ng/ml, and was decreased to 5,770 ng/ml, respectively (statistically significant according to the Friedman's test and Dunne's post-test (Figure 7).

### The Effect of the KYNA Analog, SZR72 on S100A12 (EN-RAGE) Production in the Human Whole Blood Cells of Normal Controls and of Patients With RA Stimulated by Heat-Inactivated *Staphylococcus aureus*

**Figure 8:**
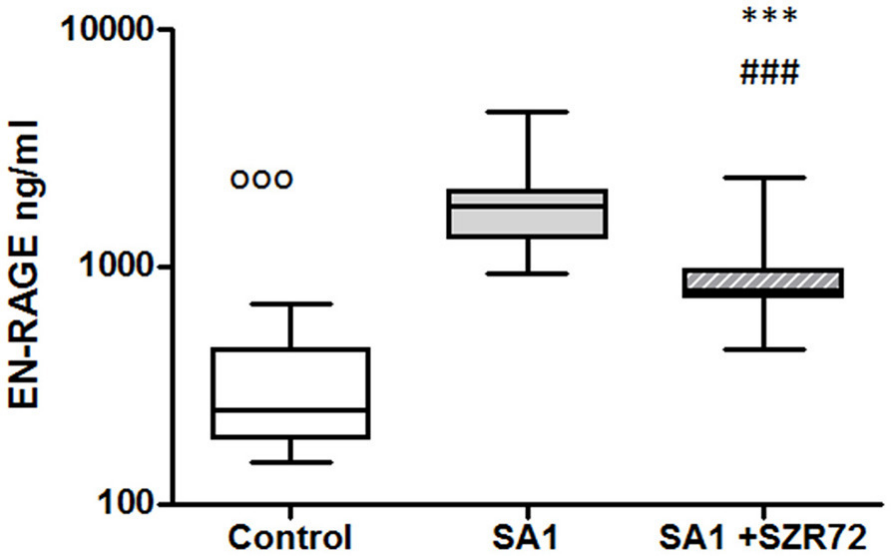
The effect of KYNA analog, SZR72 on S100A12 (EN-RAGE) production in human whole blood cells of normal blood donors stimulated by heat-inactivated *Staphylococcus aureus*. Ethylenediaminetetraacetic acid (EDTA)-anticoagulated blood samples 1–1 ml from each of 20 donors were incubated with SZR72 at a concentration of 500 μm for 30 min prior to the addition of heat-inactivated *Staphylococcus aureus* (10^7^/ml). The concentrations of EN-RAGE in the plasma were determined after an 18 h-incubation period. The data are depicted as box and whiskers plots, where the lines inside the boxes denote medians, and the boxes mark the interval between 25 and 75 percentiles, and the whiskers, the maximum and minimum. Significance was determined by the Friedman's test. Control vs. SA1, ^ooo^*p* < 0.001; control vs. SA1 + SZR 72, ^###^*p* < 0.001; SA1 vs. SA1 + SZR 72, ***p < 0.001, according to the Dunn's post-test.

**Figure 9:**
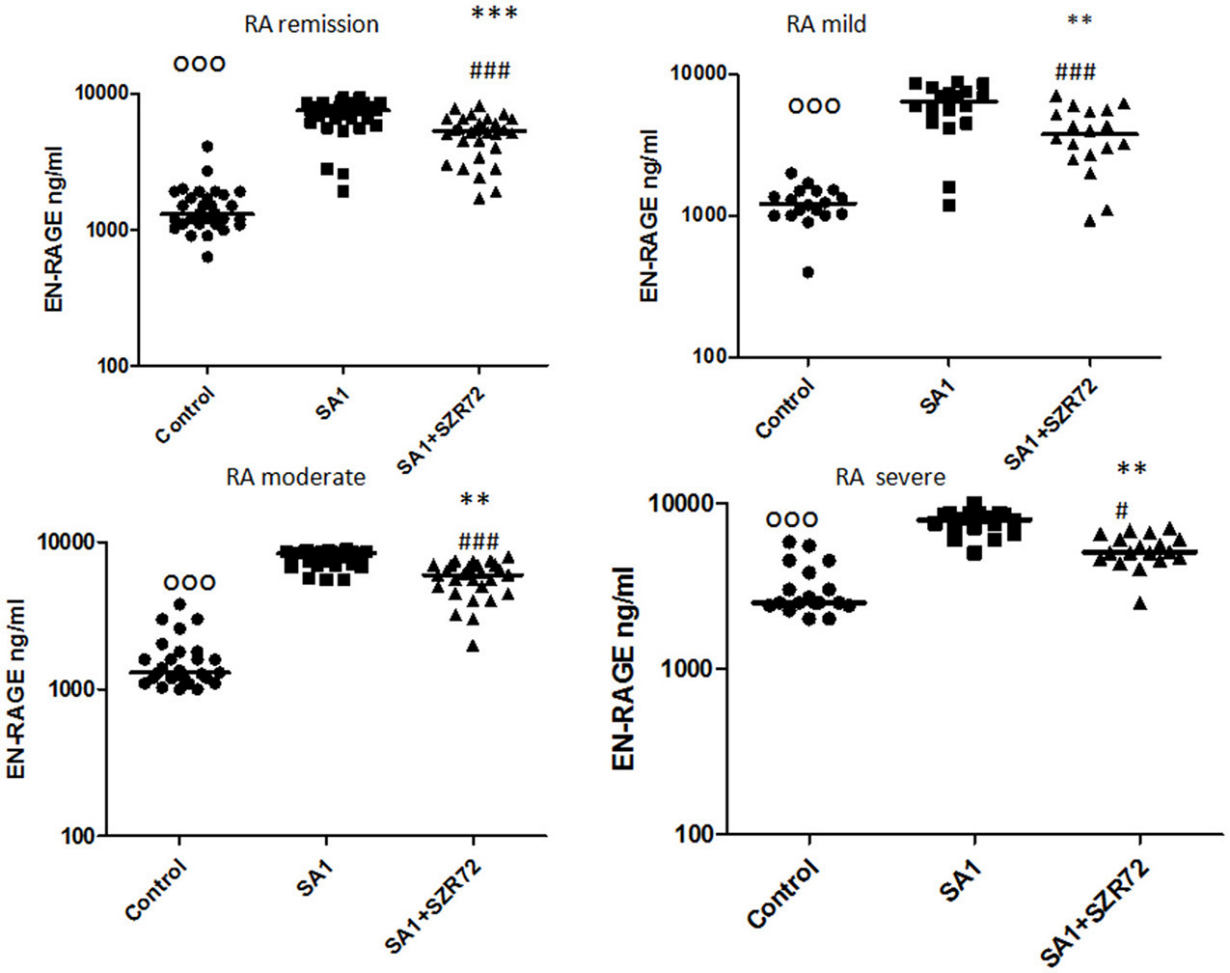
The effect of SZR72 on S100A12 (EN-RAGE) production in human whole blood cells of various groups of patients with RA stimulated by heat-inactivated *Staphylococcus aureus*. Each dot represents the individual value for one subject, and the horizontal lines denote the medians. Significance was determined by the Friedman's test. Remission group: *n* = 30, *p* < 0.0001; control vs. SA1, ^ooo^*p* < 0.001; control vs. SA1 + SZR72, ^###^*p* < 0.001; ***SA1 vs. SA1 + SZR72, *p* < 0.001 significant according to the Dunn's post-test. Mild group: *n* = 18, *p* < 0.0001; control vs. SA1, ^ooo^*p* < 0.001; control vs. SA1 + SZR72, ^###^*p* < 0.001; **SA1 vs. SA1 + SZR72, *p* < 0.01 significant with Dunn's post-test, moderate group: *n* = 27, *p* < 0.0001, control vs. SA1, ^ooo^*p* < 0.001; control vs. SA1 + SZR72, ^###^*p* < 0.001; SA1 vs. SA1 + SZR72, **p < 0.01, according to the Dunn's post-test. Severe group: *n* = 18, *p* < 0.0001; control vs. SA1 ^ooo^*p* < 0.001, control vs. SA1 + SZR72, ^#^*p* < 0.05; SA1 vs. SA1 + SZR72, **p < 0.01 according to the Dunn's post-test.

As S100A12 (EN-RAGE) is the product of mainly granulocytes, it may be of interest to investigate its level separately in patients with RA, especially the effect of the KYNA analog on the production of EN-RAGE by activated granulocytes in whole blood cultures. The concentration of S100A12 (or EN-RAGE) increased to 1,800 ng/ml in the basal levels of blood cultures of control individuals were between 150 and 700 ng/ml with the median of 250 ng/ml (Figure 8). SA1 induced the EN-RAGE production in whole blood cultures as follows: the EN-RAGE concentrations in the supernatants of SA1-induced blood cultures varied between 950 and 4,500 ng/ml, with a median of 1,800 ng/ml. At a concentration of 500 μm, SZR72 suppressed the EN-RAGE level significantly in the *S. aureus*-activated blood cultures at a median of 800 ng/ml, where *p* < 0.01 with Friedman's test and Dunn's post-test (Figure 8). We investigated whether the EN-RAGE production and the effect of SZR72 was different in the various groups of the disease. Patients were categorized according to their clinical status as mild (*n* = 18), moderate (*n* = 27), or severe (*n* = 18) status, or being in remission phase (*n* = 30) of the disease. The basal (control) median of the EN-RAGE level in 30 samples of patients in the remission phase was 1,500 ng/ml, which was elevated by SA1 up to 7,100 ng/ml. SZR72 suppressed the SA1-induced EN-RAGE production to 5,300 ng/ml, with the significance of ^***^p < 0.001 (Figure 9). The basal median level of EN-RAGE in 18 samples of the mild form of the disease was 1,215 ng/ml, which was increased in SA1-induced samples with a median of 6,400 ng/ml. SZR72 inhibited it to a median concentration of 3,750 ng/ml (Figure 9). The median level of EN-RAGE in 27 samples of the moderate group was 1,300 ng/ml, and that of the SA1-induced samples was 8,400 ng/ml, and it was suppressed by SZR72 to 6,020 ng/ml, with the significance of ^**^p < 0.01.

The EN-RAGE median level in 18 samples of patients with severe status was the highest; from 2,500 ng/ml, it was increased to 7,900 ng/ml by SA1, and decreased to 5,050 ng/ml by 500 μm SZR 72; where *p* < 0.01 according to the Friedman's test and Dunn's post-test (Figure 9).

### The Effect of the KYNA Analog, SZR72 on HNP1–3 Production in the Human Whole Blood Cells of Normal Controls and of Patients With RA Stimulated by Heat-Inactivated *Staphylococcus aureus*

**Figure 10:**
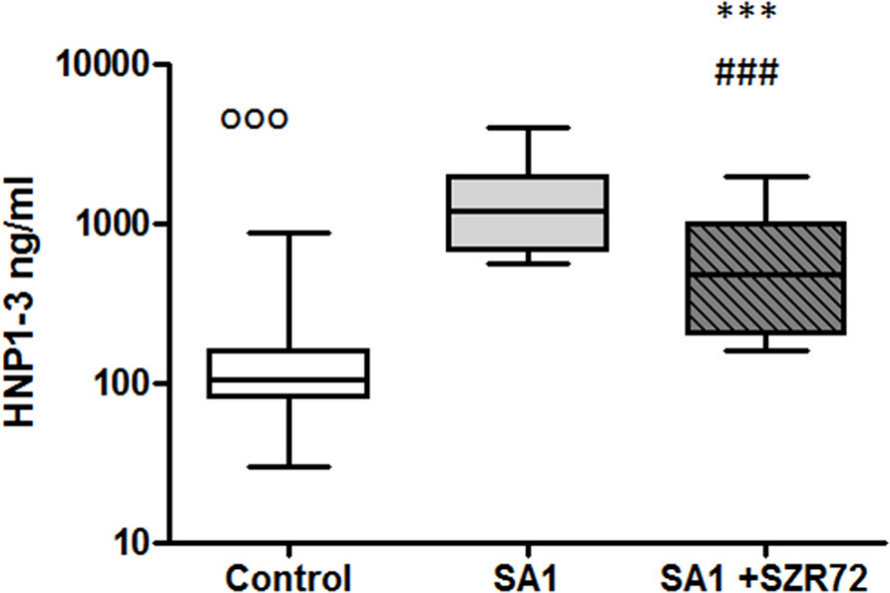
The effect of KYNA analog, SZR72 on HNP1–3 production in human whole blood cells of normal blood donors stimulated by heat-inactivated *Staphylococcus aureus*. Ethylenediaminetetraacetic acid (EDTA)-anticoagulated blood samples 1–1 ml from each of 50 healthy donors were incubated with SZR72 at a concentration of 500 μm for 30 min prior to the addition of heat-inactivated *Staphylococcus aureus* (10^7^/ml). The concentrations of HNP-1 in the plasma were determined after an 18 h-incubation period. The data are depicted as box and whiskers plots, where the lines inside the boxes denote medians, and the boxes mark the interval between 25 and 75 percentiles, and the whiskers, the maximum and minimum. Significance was determined by the Friedman's test. The values, ^ooo^*p* < 0.001 in control vs. SA1, ^###^*p* < 0.001 in control vs. SA1 + SZR72, ***p < 0.001 in SA1 + SZR72 vs. SA1.

Incubation of the whole blood of the healthy control of blood donors with heat-inactivated *S. aureus* resulted in a significant HNP-1 secretion. The median value of the basal level of HNP1–3 was 106 ng/ml. An 18 h induction by *Staphylococcus aureus* resulted in an increase in HNP1–3 level to 1,200 ng/ml. This was inhibited by the pretreatment with 500 um SZR72 to a median of 475 ng/ml (*p* < 0.001; Figure 10) for the comparison; altogether, 89 blood samples of patients with RA were processed for measuring HNP1–3 by ELISA.

**Figure 11:**
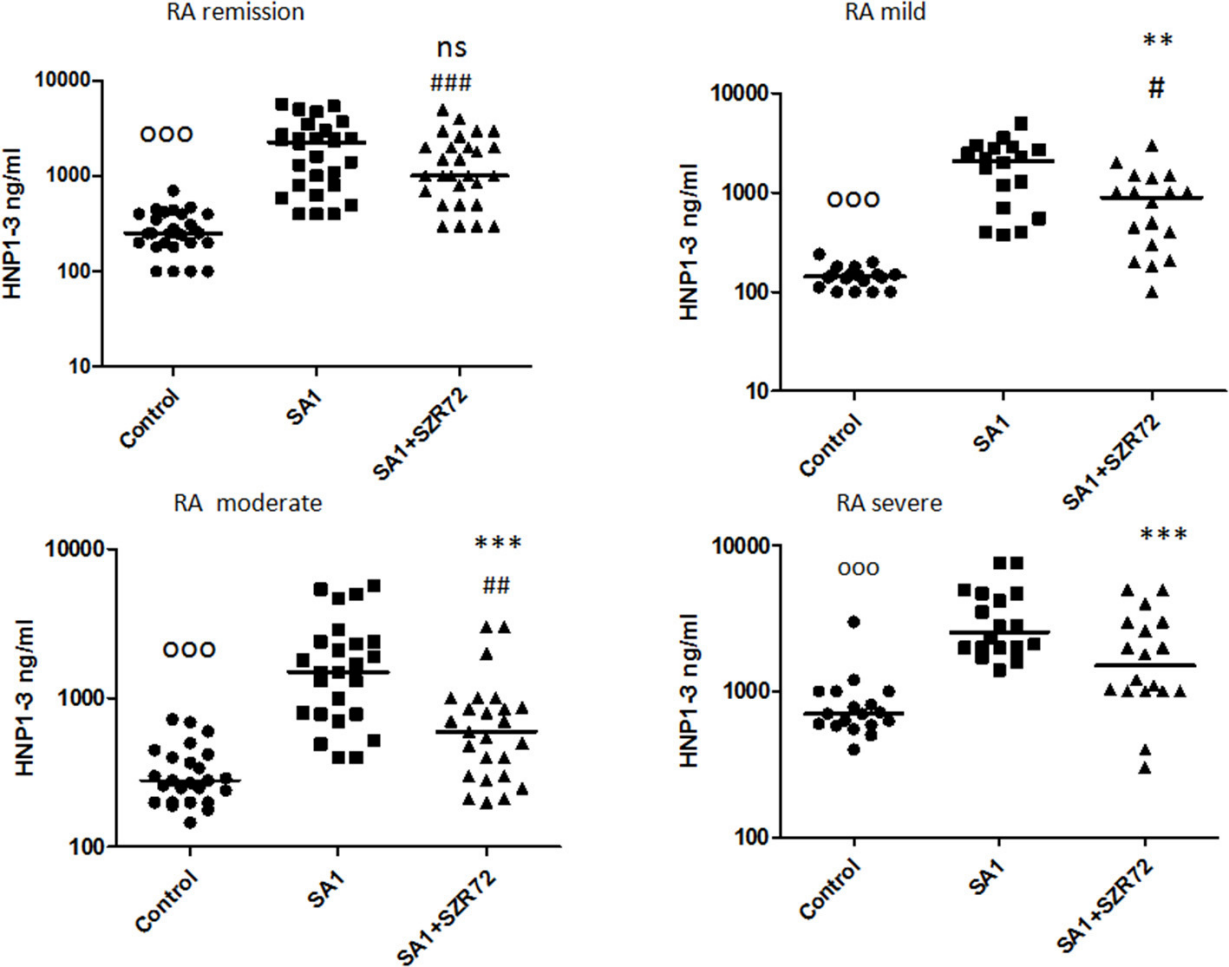
The effect of SZR72 on HNP1–3 production in human whole blood cells of various groups of patients with RA stimulated by heat-inactivated *Staphylococcus aureus*. Each dot represents the individual value for one subject, and the horizontal lines denote the medians. Significance was determined by the Friedman's test. Remission group: *n* = 28, *p* < 0.0001; control vs. SA1, ^ooo^*p* < 0.001; control vs. SA1 + SZR72, ^###^*p* < 0.001; SA1 vs. SA1 + SZR72, non-significant, according to the Dunn's post-test. Mild group: *n* = 18, *p* < 0.0001, control vs. SA1, ^ooo^*p* < 0.001; control vs. SA1 + SZR72, ^#^*p* < 0.05, SA1 vs. SA1 + SZR72, **p < 0.01 is significant with Dunn's post-test; moderate group: *n* = 25, *p* < 0.0001; control vs. SA1, ^ooo^*p* < 0.001; control vs. SA1 + SZR72, ^##^*p* < 0.01; SA1 vs. SA1 + SZR72, ***p < 0.001 significant according to the Dunn's post-test. Severe group: *n* = 18, *p* < 0.0001; ^ooo^*p* < 0.001; control vs. SA1, significant, control vs. SA1 + SZR72, non-significant, ***SA1 vs. SA1 + SZR72, *p* < 0.001 significant according to the Dunn's post-test.

We stratified the patients into different groups according to their clinical status: 18 patients with the mild form of disease, 18 patients with the severe form of the disease, 25 patients with the intermediate form of the disease, and 28 patients with the remission phase of RA. The basal (control) median HNP1–3 level in 28 samples of patients with the remission phase was 250 ng/ml, which was elevated by SA1 up to 2,250 ng/ml. SZR72 suppressed the SA1-induced EN-RAGE production to 1,000 ng/ml; however, this was not statistically significant (Figure 11). The HNP1–3 concentration varied from 100 to 240 ng/ml in the whole blood samples of patients in the mild group (Figure 11). SA1 enhanced HNP1–3 secretion to 2,100 ng/ml as median, which was suppressed to 900 ng/ml by SZR72 (*p* < 0.01, Figure 11). When 25 samples from the moderate group were analyzed, the median level of HNP1–3 was 280 ng/ml, and the increase in its secretion was observed following SA1 induction to 1,500 ng/ml, and SZR72 significantly (*p* < 0.001) reduced it to 600 ng/ml as a median, which was also significant according to Friedman's test and Dunn's post-test (Figure 11). The median basal level of HNP1–3 in samples of patients having a severe form of the disease was the highest, reaching 700 ng/ml. These high HNP1–3 levels might be the consequence of the degranulation of recruited neutrophils. SA1 activation resulted in 2,550 ng/ml, and the SZR72 treatment resulted in a decrease to 1,500 ng/ml, which was statistically significant (*p* < 0.001, Figure 11).

### Comparison of the Effect of SZR72 on the Blood of Patients With RA Divided According to Seropositivity and Medication

**Figure 12:**
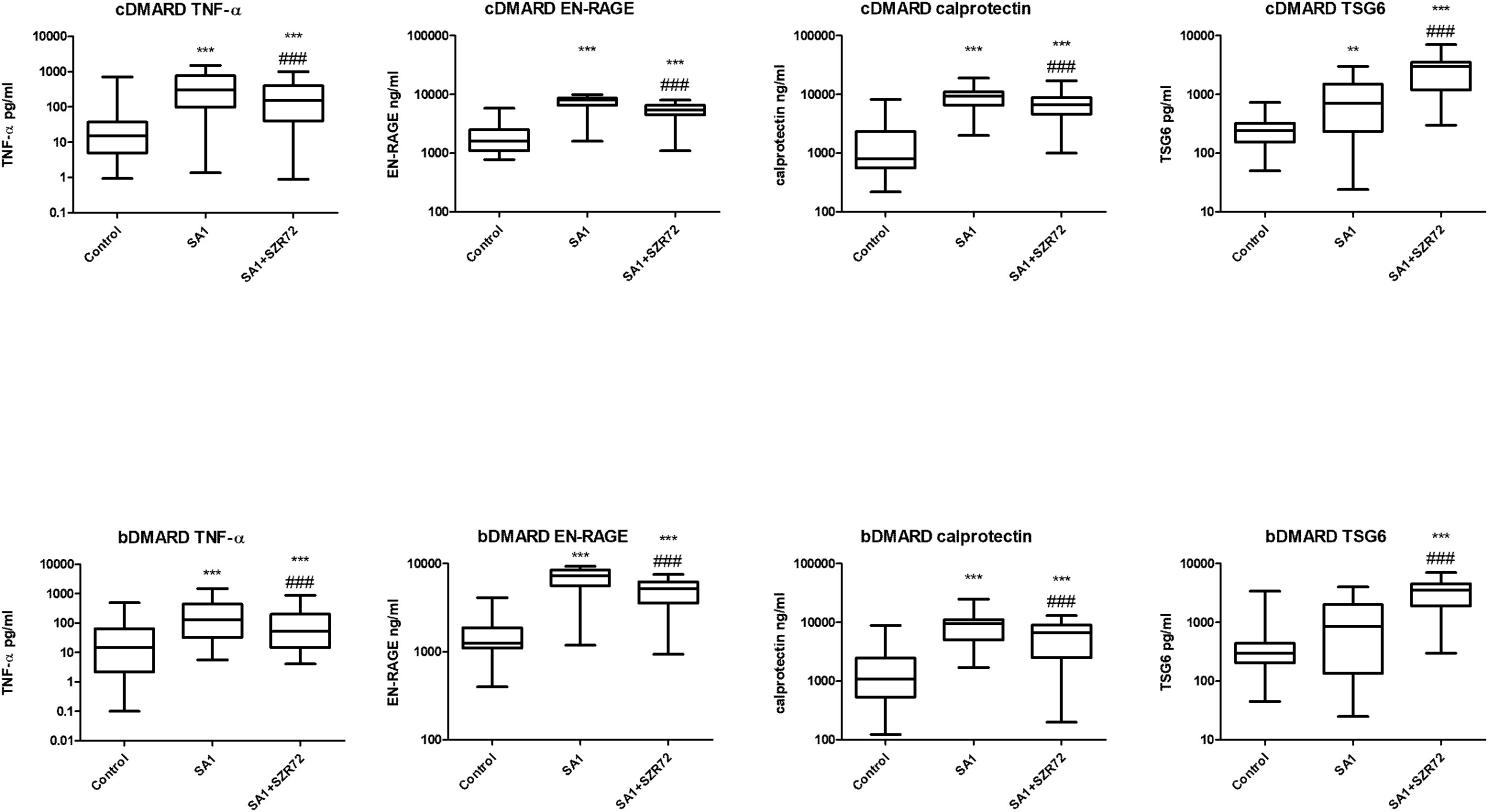
Levels of tumor necrosis factor-α (TNF-α), EN-RAGE, calprotectin, and TSG-6 at baseline and following treatments with SA1 and SA1 + SZR72 in patients with RA on conventional disease modifying anti-rheumatic drugs (cDMARD, *n* = 41) or on biologic disease modifying anti-rheumatic drugs (bDMARD, *n* = 41). Comparisons were made with the Friedman's test, where **p < 0.01 vs. control, ***p < 0.001 vs. control, and ^###^*p* < 0.001 vs. SA1. Horizontal line—median, box—interquartile range, whiskers—range.

**Figure 13:**
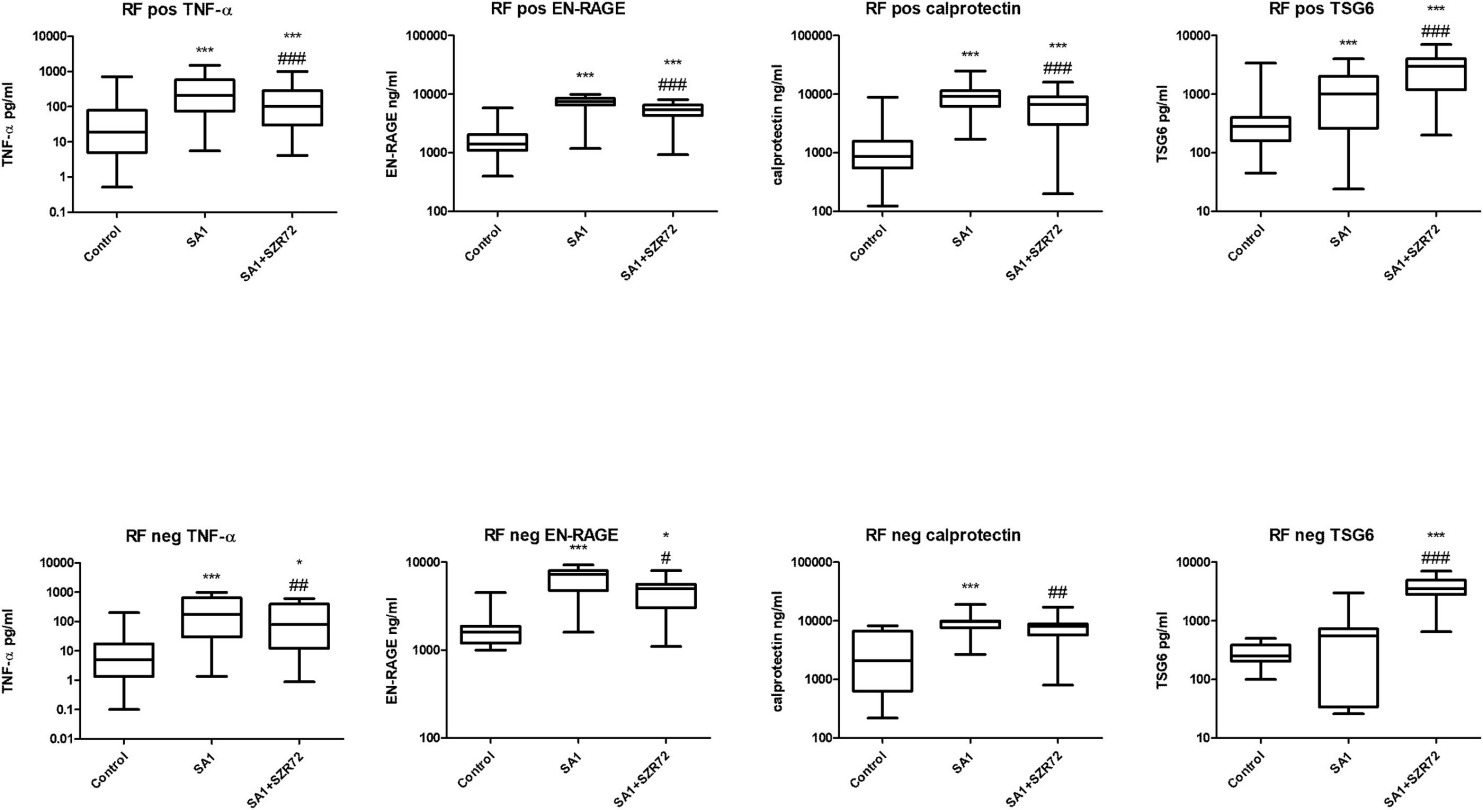
Levels of TNF- α, EN-RAGE, calprotectin, and TSG-6 at baseline and following treatment with SA1 and SA1 + SZR72 in rheumatoid factor (RF) in patients with positive (*n* = 72) and negative rheumatoid arthritis (RA) (*n* = 17). Comparisons were made with Friedman's tests *p < 0.5 vs. control, ***p < 0.001 vs. control, ^#^*p* < 0.05 vs. SA1, ^##^*p* < 0.01 vs. SA1, ^###^*p* < 0.001 vs. SA1. Horizontal line—median, box—interquartile range, whiskers—range.

Both medication and RF status would influence on the investigated parameters. Therefore, we compared the different patient groups according to their medication (Figure 12). There was no significant difference between the patients treated with cDMARD (conventional) vs. bDMARD (biological therapy) (Figure 12). Similarly, there was no different tendency in response on the KYNA analog based on the RF status of the patients (Figure 13).

## Conclusion

This is the first complex study to investigate the effects of KYNA analog, SZR72 on the *in vitro* production of TNF-α, TSG-6, and the calgranulins as calprotectin and EN-RAGE, together with HNP1–3 (defensin-α) in patients with RA in various activities of the disease.

In the whole blood model, which resembles closely the physiological milieu, the KYNA analog, SZR72 inhibited the production not only of TNF-α, but also of calprotectin, EN-RAGE, and HNP1–3 in the *in vitro* activated blood cultures of patients with RA.

Granulocytic defensin-α secretion was measured in our experiments also in whole blood cultures. Since granulocytes are the main source of HNP1–3 in the peripheral blood, the observations obtained from the investigation of whole blood may be regarded as reflective of the granulocyte functions (37, 38). Our previous experiments revealed that HNP1–3 was secreted not only by purified granulocytes but also in the case of the whole blood incubation method (20, 37). As mentioned, neutrophil granulocytes are the main source of HNP1–3; therefore, we suggest that the increase of HNP1–3 levels following SA1 induction mostly originates from granulocyte activity. Accordingly, we infer that the calgranulins in the supernatants of whole blood cultures might be regarded as products partially of monocytes and mainly of granulocytes (27, 28, 39).

There are previous data indicating the role of these inflammatory mediators in the pathogenesis of RA. Especially, TNF-α has a pivotal role in the exaggerated cytokine activation; therefore, the therapeutic use of anti-TNF as a biological response modifier has a pioneering importance in RA (4, 5). Higher levels of calprotectin (24, 25, 40, 41), HNP1–3, and EN-RAGE in sera of patients with RA have been detected in some clinical studies (32–34).

In contrast, in our study, lower TSG-6 levels were detected in the severe group of patients with RA compared to healthy controls (Table 2). In the present study, the effect of the KYNA analog was investigated on the TSG-6 production *ex vivo* in human whole blood cultures of patients with RA. The KYNA analog. SZR72 exerted a stimulatory effect on the TSG-6 production in the whole blood cultures of patients with RA in various activities of the disease. We hypothesize that it contributes to the anti-inflammatory effect of the drug by inhibiting the TNF-α synthesis (23, 42, 43). The beneficial role of TSG-6 has been interpreted as inhibiting the association of TLR4 with MyD88, thereby suppressing NF-κB activation (43). TSG-6 has also prevented the expression of proinflammatory proteins (iNOS, IL-6, TNFα, and IL-1β) (42), and its expression might be under the influence of KYNA (44). Thus, we suppose that increasing the production of TSG-6 by the KYNA analog might contribute to the inhibitory effect toward TNF-α synthesis in human blood cultures. The opposite effects of the KYNA analogs on TNF-α and TSG-6 have been detected previously in cell culture experiments (23).

**Table 2:** Levels of inflammatory mediators in the plasma of RA patients and in healthy donors.

	**Healthy individuals *n* = 50**	**RA remission *n* = 30 (28+)**	**RA mild *n* = 18**	**RA moderate *n* = 27 (25+)**	**RA active *n* = 18**
TNF-α pg/mL	4.35 [1.94–15]	5.0 [1.8–15.0]	6.5 [2.1–12.75]	15 [2.5–49.5]	85 [19.5–4,000]^***^
TSG-6 pg/mL	340 [237–510]	400 [307–500]	445 [257–537]	260 [200–310]^*^	150 [59–210]^***^
Calprotectin ng/mL	500 [220–712]	911 [648–1,420]^***^	680 [345–861]^ns^	1,056 [560–6,390]^**^	6,700 [5,713–7,716]^***^
EN-RAGE ng/mL	250 [187–450]	1,300 [1,100–1,825]^***^	1,215 [1,000–1,500]^*^	1,300 [1,190–1,800]^***^	2,500 [2,400–5,800]^***^
HNP1–3 ng/mL (+)	106 [80–160]	250 [53–999]^***^	143 [109–157]^ns^	280 [200–410]^***^	700 [587–1,000]^***^

*Data are expressed as median [interquartile range]. Comparisons were made using the Kruskal–Wallis test*.

*** p < 0.001 vs. healthy individuals,

** p < 0.01 vs healthy individuals,

* *p < 0.05 vs healthy individuals, ns, non-significant, (+): 89 test of RA patients*.

*EN-RAGE, extracellular newly identified receptor for advanced glycation end-products binding protein; RA, rheumatoid arthritis; TNF-α, Tumor Necrosis Factor-α; TSG-6, Tumor Necrosis Factor-Stimulated Gene-6*.

In our experiments, SZR72 inhibited the production of the inflammatory mediators in whole blood cultures, which were induced by heat-inactivated SA1. This effect was observed in all groups of patients in the various activities of the disease. It is also noteworthy that the basic (control) levels of these mediators were higher in the blood of patients than in healthy donors (Table 2). When we compared the data of patients with that of healthy individuals (*n* = 50), the levels of TNF-α, calprotectin, EN-RAGE, HNP1–3 were lower compared to the data of all patients with RA. These data are in good correlation with the observations, concluding that these inflammatory mediators are higher in patients with RA (4, 5, 25, 32–34, 41). Inhibition of their secretion can, therefore, additionally result in an anti-inflammatory effect in RA.

In inflamed tissues, calprotectins (S100A8 and S100A9) have a potential role as a target of treatment in murine models of autoimmune disorders, since the direct or indirect blockade of these proteins result in amelioration of the disease process (45).

In our experiments, the basal levels of these mediators were further increased following the activation of the blood cells, but the KYNA analog significantly reduced their production from the activated cells.

It was of interest to observe whether normal (healthy) control cells subjected to Staphylococcus antigen with or without SZR72 would react similarly or in a different way than cells from patients with RA. Therefore, in a pilot study, we performed similar *ex vivo* experiments with the 50 normal blood donors (Figures 2, 4, 6, 8, 10). On the basis of these results, we conclude that SZR72 at 500 μm exerted a significant inhibitory effect on TNF-α, calprotectin, EN-RAGE, and HNP-1–3 production on SA1-stimulated whole blood, and increased the TSG-6 production.

We compared the different patient groups according to their medication (Figure 12). There was no significant difference between the patients treated with cDMARD vs. bDMARD. Similarly, there was no different tendency in response to the KYNA analog based on RF status of the patients (Figure 13).

Our study has limitations. According to our previous results (20, 23), 500 μm of KYNA analog was used. The median level of KYNA in human sera is at nanomolar (30–40 nm) range of concentration (6, 46, 47). However, it increases considerably in the course of infections and inflammatory processes due to increased degradation of tryptophan. In addition, during different *in vitro* experiments, micromolar concentrations were found to be effective (44, 47). High micromolar concentrations of KYNA are able to block NMDA receptor function (47). Moreover, we have to take into consideration that in our study, a potential therapeutic effect was investigated. Another question is the duration of the incubation period of the whole blood, that is, 18 h. For the determination of the effect of SA1 and the KYNA analog on the cytokine-protein production, it was considered to be necessary. Similarly, relatively long or even longer incubation periods were applied in experiments with mice splenocytes (48), or with human mesenchymal cells (44). In another study (6), human mononuclear cells or human monocytes were treated with KYNA at 0.5–1 mm for 18 h for the determination of TNF-α in cell culture supernatants following lipopolysaccharide (LPS) induction. The potential degradation of SZR72 to KYNA in our experiments was not investigated, but it cannot be excluded (15, 49–51).

Taken together, these data further support the immunomodulatory role of KYNA in RA (52), and may draw the attention to the importance of the synthesis of the KYNA analog, which might have a future therapeutic potential. Therefore, we suggest that these experiments further explore the potential benefits of the future application of the KYNA analogs in RA.

## Data Availability Statement

The raw data supporting the conclusions of this article will be made available by the authors, without undue reservation.

## Ethics Statement

The project was approved by the Ethics Committee of the University of Szeged (ETT-TUKEB 905/PI/09 and 149/2019-SZOTE). This study was conducted in full accordance with the Declaration of Helsinki (1964). The patients/participants provided their written informed consent to participate in this study.

## Author Contributions

AB conducted the clinical management of patients and supervised the study. BV performed the experiments with the blood samples. FF elaborated SZR-72. IL analyzed the data. GT supervised the new statistical methods and added new figures and tables. LV organized the research for the neurological project. YM designed the research and prepared the manuscript. All authors have read the manuscript and approved it.

## Conflict of Interest

The authors declare that the research was conducted in the absence of any commercial or financial relationships that could be construed as a potential conflict of interest.

## References

[1] Firestein GS McInnes IB. Immunopathogenesis of rheumatoid arthritis. Immunity. (2017) 46:183–96. 10.1016/j.immuni.2017.02.006 28228278 PMC5385708

[2] Smolen JS Aletaha D Barton A Burmester GR Emery P Firestein GS. Rheumatoid arthritis. Nat Rev Dis Primer. (2018) 4:18001. 10.1038/nrdp.2018.1 29417936

[3] Mateen S Zafar A Moin S Khan AQ Zubair S. Understanding the role of cytokines in the pathogenesis of rheumatoid arthritis. Clin Chim Acta Int J Clin Chem. (2016) 455:161–71. 10.1016/j.cca.2016.02.010 26883280

[4] Maini RN Brennan FM Williams R Chu CQ Cope AP Gibbons D. TNF-alpha in rheumatoid arthritis and prospects of anti-TNF therapy. Clin Exp Rheumatol. (1993) 11(Suppl. 8):S173–5. 8391952

[5] Radner H Aletaha D. Anti-TNF in rheumatoid arthritis: an overview. Wien Med Wien Med Wochenschr. (2015) 165:3–9. 10.1007/s10354-015-0344-y 25651945

[6] Parada-Turska J Zgrajka W Majdan M. Kynurenic acid in synovial fluid and serum of patients with rheumatoid arthritis, spondyloarthropathy, and osteoarthritis. J Rheumatol. (2013) 40:903–9. 10.3899/jrheum.121035 23588943

[7] Ogbechi J Clanchy FI Huang Y-S Topping LM Stone TW Williams RO. IDO activation, inflammation and musculoskeletal disease. Exp Gerontol. (2020) 131:110820. 10.1016/j.exger.2019.110820 31884118

[8] Swartz KJ During MJ Freese A Beal MF. Cerebral synthesis and release of kynurenic acid: an endogenous antagonist of excitatory amino acid receptors. J Neurosci. (1990) 10:2965–73. 10.1523/JNEUROSCI.10-09-02965.1990 2168940 PMC6570241

[9] Vécsei L Miller J MacGarvey U Beal MF. Kynurenine and probenecid inhibit pentylenetetrazol- and NMDLA-induced seizures and increase kynurenic acid concentrations in the brain. Brain Res Bull. (1992) 28:233–8. 10.1016/0361-9230(92)90184-Y 1596743

[10] Hilmas C Pereira EF Alkondon M Rassoulpour A Schwarcz R Albuquerque EX. The brain metabolite kynurenic acid inhibits alpha7 nicotinic receptor activity and increases non-alpha7 nicotinic receptor expression: physiopathological implications. J Neurosci. (2001) 21:7463–73. 10.1523/JNEUROSCI.21-19-07463.2001 11567036 PMC6762893

[11] Stone TW. Kynurenic acid blocks nicotinic synaptic transmission to hippocampal interneurons in young rats. Eur J Neurosci. (2007) 25:2656–65. 10.1111/j.1460-9568.2007.05540.x 17459105

[12] Stone TW. Development and therapeutic potential of kynurenic acid and kynurenine derivatives for neuroprotection. Trends Pharmacol Sci. (2000) 21:149–54. 10.1016/S0165-6147(00)01451-6 10740291

[13] Robotka H Toldi J Vécsei L. L-kynurenine: metabolism and mechanism of neuroprotection. Future Neurol. (2008) 3:169–88. 10.2217/14796708.3.2.169

[14] Mándi Y Vécsei L. The kynurenine system and immunoregulation. J Neural Transm. (2012) 119:197–209. 10.1007/s00702-011-0681-y 21744051

[15] Wirthgen E Hoeflich A Rebl A Günther J. Kynurenic acid: the janus-faced role of an immunomodulatory tryptophan metabolite and its link to pathological conditions. Front Immunol. (2017) 8:1957. 10.3389/fimmu.2017.01957 29379504 PMC5770815

[16] Wang J Simonavicius N Wu X Swaminath G Reagan J Tian H. Kynurenic acid as a ligand for orphan G protein-coupled receptor GPR35. J Biol Chem. (2006) 281:22021–8. 10.1074/jbc.M603503200 16754668

[17] Julliard W Fechner JH Mezrich JD. The aryl hydrocarbon receptor meets immunology: friend or foe? A little of both. Front Immunol. (2014) 5:458. 10.3389/fimmu.2014.00458 25324842 PMC4183121

[18] Fülöp F Szatmári I Vámos E Zádori D Toldi J Vécsei L. Syntheses, transformations and pharmaceutical applications of kynurenic acid derivatives. Curr Med Chem. (2009) 16:4828–42. 10.2174/092986709789909602 19929784

[19] Marosi M Nagy D Farkas T Kis Z Rózsa E Robotka H. A novel kynurenic acid analogue: a comparison with kynurenic acid. An *in vitro* electrophysiological study. J Neural Transm. (2010) 117:183–8. 10.1007/s00702-009-0346-2 19953278

[20] Tiszlavicz Z Németh B Fülöp F Vécsei L Tápai K Ocsovszky I. Different inhibitory effects of kynurenic acid and a novel kynurenic acid analogue on tumour necrosis factor-α (TNF-α) production by mononuclear cells, HMGB1 production by monocytes and HNP1-3 secretion by neutrophils. Naunyn Schmiedebergs Arch Pharmacol. (2011) 383:447–55. 10.1007/s00210-011-0605-2 21336543

[21] Fülöp F Szatmári I Toldi J Vécsei L. Modifications on the carboxylic function of kynurenic acid. J Neural Transm. (2012) 119:109–14. 10.1007/s00702-011-0721-7 21997444

[22] Juhász L Rutai A Fejes R Tallósy SP Poles MZ Szabó A. Divergent effects of the N-methyl-D-aspartate receptor antagonist kynurenic acid and the synthetic analog SZR-72 on microcirculatory and mitochondrial dysfunction in experimental sepsis. Front Med. (2020) 7:566582. 10.3389/fmed.2020.566582 33330526 PMC7729001

[23] Mándi Y Endrész V Mosolygó T Burián K Lantos I Fülöp F. The opposite effects of kynurenic acid and different kynurenic acid analogs on tumor necrosis factor-α (TNF-α) production and tumor necrosis factor-stimulated gene-6 (TSG-6) expression. Front Immunol. (2019) 10:1406. 10.3389/fimmu.2019.01406 31316502 PMC6611419

[24] Ometto F Friso L Astorri D Botsios C Raffeiner B Punzi L. Calprotectin in rheumatic diseases. Exp Biol Med Maywood NJ. (2017) 242:859–73. 10.1177/1535370216681551 PMC5407536 27895095

[25] Bae S-C Lee YH. Calprotectin levels in rheumatoid arthritis and their correlation with disease activity: a meta-analysis. Postgrad Med. (2017) 129:531–7. 10.1080/00325481.2017.1319729 28425837

[26] Foell D Wittkowski H Vogl T Roth J. S100 proteins expressed in phagocytes: a novel group of damage-associated molecular pattern molecules. J Leukoc Biol. (2007) 81:28–37. 10.1189/jlb.0306170 16943388

[27] Pietzsch J Hoppmann S. Human S100A12: a novel key player in inflammation? Amino Acids. (2009) 36:381–9. 10.1007/s00726-008-0097-7 18443896

[28] Wang S Song R Wang Z Jing Z Wang S Ma J. S100A8/A9 in inflammation. Front Immunol. (2018) 9:1298. 10.3389/fimmu.2018.01298 29942307 PMC6004386

[29] Ganz T. Extracellular release of antimicrobial defensins by human polymorphonuclear leukocytes. Infect Immun. (1987) 55:568–71. 10.1128/IAI.55.3.568-571.1987 3643886 PMC260375

[30] Quinn K Henriques M Parker T Slutsky AS Zhang H. Human neutrophil peptides: a novel potential mediator of inflammatory cardiovascular diseases. Am J Physiol Heart Circ Physiol. (2008) 295:H1817–24. 10.1152/ajpheart.00472.2008 18805897 PMC4896811

[31] Yang D Biragyn A Kwak LW Oppenheim JJ. Mammalian defensins in immunity: more than just microbicidal. Trends Immunol. (2002) 23:291–6. 10.1016/S1471-4906(02)02246-9 12072367

[32] Bokarewa MI Jin T Tarkowski A. Intraarticular release and accumulation of defensins and bactericidal/permeability-increasing protein in patients with rheumatoid arthritis. J Rheumatol. (2003) 30:1719–24. 12913926

[33] Okcu M Oktayoglu P Mete N Bozkurt M Caglayan M Dagli AZ. A useful marker in the assessment of remission and activation of disease in patients with rheumatoid arthritis: serum human neutrophil peptides 1-3. J Back Musculoskelet Rehabil. (2018) 31:1145–50. 10.3233/BMR-160743 29991120

[34] Chen Y-S Yan W Geczy CL Brown MA Thomas R. Serum levels of soluble receptor for advanced glycation end products and of S100 proteins are associated with inflammatory, autoantibody, and classical risk markers of joint and vascular damage in rheumatoid arthritis. Arthritis Res Ther. (2009) 11:R39. 10.1186/ar2645 19284577 PMC2688185

[35] Kay J Upchurch KS. ACR/EULAR 2010 rheumatoid arthritis classification criteria. Rheumatol Oxf Engl. (2012) 51(Suppl. 6), vi5–vi9. 10.1093/rheumatology/kes279 23221588

[36] Wang JE Jørgensen PF Almlöf M Thiemermann C Foster SJ Aasen AO. Peptidoglycan and lipoteichoic acid from Staphylococcus aureus induce tumor necrosis factor alpha, interleukin 6 (IL-6), and IL-10 production in both T cells and monocytes in a human whole blood model. Infect Immun. (2000) 68:3965–70. 10.1128/IAI.68.7.3965-3970.2000 10858210 PMC101674

[37] Kocsis AK Ocsovszky I Tiszlavicz L Tiszlavicz Z Mándi Y. Helicobacter pylori induces the release of alpha-defensin by human granulocytes. Inflamm Res. (2009) 58:241–7. 10.1007/s00011-008-8100-z 19169650

[38] Sthoeger ZM Bezalel S Chapnik N Asher I Froy O. High α-defensin levels in patients with systemic lupus erythematosus. Immunology. (2009) 127:116–22. 10.1111/j.1365-2567.2008.02997.x 19191901 PMC2678187

[39] Roth J Vogl T Sorg C Sunderkötter C. Phagocyte-specific S100 proteins: a novel group of proinflammatory molecules. Trends Immunol. (2003) 24:155–8. 10.1016/S1471-4906(03)00062-0 12697438

[40] Hammer HB Odegard S Fagerhol MK Landewé R van der Heijde D Uhlig T. Calprotectin (a major leucocyte protein) is strongly and independently correlated with joint inflammation and damage in rheumatoid arthritis. Ann Rheum Dis. (2007) 66:1093–7. 10.1136/ard.2006.064741 17234650 PMC1954700

[41] Wang Q Chen W Lin J. The role of calprotectin in rheumatoid arthritis. J Transl Intern Med. (2019) 7:126–31. 10.2478/jtim-2019-0026 PMC6985918 32010597

[42] Day AJ Milner CM. TSG-6: a multifunctional protein with anti-inflammatory and tissue-protective properties. Matrix Biol J Int Soc Matrix Biol. (2019) 78–9:60–83. 10.1016/j.matbio.2018.01.011 29362135

[43] Choi H Lee RH Bazhanov N Oh JY Prockop DJ. Anti-inflammatory protein TSG-6 secreted by activated MSCs attenuates zymosan-induced mouse peritonitis by decreasing TLR2/NF-κB signaling in resident macrophages. Blood. (2011) 118:330–8. 10.1182/blood-2010-12-327353 21551236 PMC3138686

[44] Wang G Cao K Liu K Xue Y Roberts AI Li F. Kynurenic acid, an IDO metabolite, controls TSG-6-mediated immunosuppression of human mesenchymal stem cells. Cell Death Differ. (2018) 25:1209–23. 10.1038/s41418-017-0006-2 29238069 PMC6030103

[45] Pruenster M Vogl T Roth J Sperandio M. S100A8/A9: from basic science to clinical application. Pharmacol Ther. (2016) 167:120–31. 10.1016/j.pharmthera.2016.07.015 27492899

[46] Sellgren CM Gracias J Jungholm O Perlis RH Engberg G Schwieler L. Peripheral and central levels of kynurenic acid in bipolar disorder subjects and healthy controls. Transl Psychiatry. (2019) 9:37. 10.1038/s41398-019-0378-9 30696814 PMC6351610

[47] Stone TW. Neuropharmacology of quinolinic and kynurenic acids. Pharmacol Rev. (1993) 45:309–79. 8248282

[48] Małaczewska J Siwicki AK Wójcik RM Turski WA Kaczorek E. The effect of kynurenic acid on the synthesis of selected cytokines by murine splenocytes - *in vitro* and *ex vivo* studies. Cent Eur J Immunol. (2016) 41:39–46. 10.5114/ceji.2016.58815 27095921 PMC4829820

[49] Zádori D Ilisz I Klivényi P Szatmári I Fülöp F Toldi J. Time-course of kynurenic acid concentration in mouse serum following the administration of a novel kynurenic acid analog. J Pharm Biomed Anal. (2011) 55:540–3. 10.1016/j.jpba.2011.02.014 21392919

[50] Fejes-Szabó A Bohár Z Vámos E Nagy-Grócz G Tar L Veres G. Pre-treatment with new kynurenic acid amide dose-dependently prevents the nitroglycerine-induced neuronal activation and sensitization in cervical part of trigemino-cervical complex. J Neural Transm. (2014) 121:725–38. 10.1007/s00702-013-1146-2 24385076

[51] Fehér E Szatmári I Dudás T Zalatnai A Farkas T Lorinczi B. Structural evaluation and electrophysiological effects of some kynurenic acid analogs. Mol Basel Switz. (2019) 24:3502. 10.3390/molecules24193502 31561643 PMC6803921

[52] Huang Y-S Ogbechi J Clanchy FI Williams RO Stone TW. IDO and kynurenine metabolites in peripheral and CNS disorders. Front Immunol. (2020) 11:388. 10.3389/fimmu.2020.0038 32194572 PMC7066259

